# A century of BCG vaccination: Immune mechanisms, animal models, non-traditional routes and implications for COVID-19

**DOI:** 10.3389/fimmu.2022.959656

**Published:** 2022-08-26

**Authors:** Shivani Singh, Noemi Alejandra Saavedra-Avila, Sangeeta Tiwari, Steven A. Porcelli

**Affiliations:** ^1^ Department of Medicine, New York University School of Medicine, New York, NY, United States; ^2^ Department of Microbiology and Immunology, Albert Einstein College of Medicine, New York, NY, United States; ^3^ Department of Medicine, Albert Einstein College of Medicine, New York, NY, United States; ^4^ Department of Biological Sciences and Border Biomedical Research Center, University of Texas at El Paso, Texas, United States

**Keywords:** tuberculosis, vaccine, immunity, trained immunity, recombinant BCG

## Abstract

Bacillus Calmette-Guerin (BCG) has been used as a vaccine against tuberculosis since 1921 and remains the only currently approved vaccine for this infection. The recent discovery that BCG protects against initial infection, and not just against progression from latent to active disease, has significant implications for ongoing research into the immune mechanisms that are relevant to generate a solid host defense against *Mycobacterium tuberculosis* (Mtb). In this review, we first explore the different components of immunity that are augmented after BCG vaccination. Next, we summarize current efforts to improve the efficacy of BCG through the development of recombinant strains, heterologous prime-boost approaches and the deployment of non-traditional routes. These efforts have included the development of new recombinant BCG strains, and various strategies for expression of important antigens such as those deleted during the *M. bovis* attenuation process or antigens that are present only in Mtb. BCG is typically administered *via* the intradermal route, raising questions about whether this could account for its apparent failure to generate long-lasting immunological memory in the lungs and the inconsistent level of protection against pulmonary tuberculosis in adults. Recent years have seen a resurgence of interest in the mucosal and intravenous delivery routes as they have been shown to induce a better immune response both in the systemic and mucosal compartments. Finally, we discuss the potential benefits of the ability of BCG to confer trained immunity in a non-specific manner by broadly stimulating a host immunity resulting in a generalized survival benefit in neonates and the elderly, while potentially offering benefits for the control of new and emerging infectious diseases such as COVID-19. Given that BCG will likely continue to be widely used well into the future, it remains of critical importance to better understand the immune responses driven by it and how to leverage these for the design of improved vaccination strategies against tuberculosis.

## 1 Introduction

Tuberculosis (TB) continues to be a global health catastrophe and kills over a million people annually (1). Prolonged treatment regimens and poor drug compliance have led to the emergence of drug resistant strains, further complicating the approach to this pandemic. The attenuated *Mycobacterium bovis* (*M. bovis*) Bacille Calmette-Guerin (BCG) strain has been in use as a vaccine since 1921 and is the only currently approved vaccine for TB (2). It was initially obtained from a virulent strain of *M. bovis* that was attenuated following ~230 passages *in vitro*. BCG has immuno-modulatory effects that make it effective against central nervous and disseminated TB when administered at birth or to school age children, but has shown minimal or variable protection against adult pulmonary TB (3). Much of the poor efficacy of BCG has been attributed to the attenuation process, that led to multiple genomic deletions resulting in 16 genomic regions of difference (RD1 through RD16) compared to the *Mycobacterium tuberculosis* (Mtb) genome (4). Despite these concerns and limitations, BCG has an established safety profile and is on the immunization program of several high TB burden countries, making it one of the most widely used vaccines in the world today. Several pre-clinical vaccine development studies and clinical trials have demonstrated that as a stand-alone vaccine, BCG is at least as efficacious as any of the newer subunit TB vaccines tested to date (5). Several TB vaccination trials are therefore striving to retain the protective benefits of BCG through the development and study of recombinant strains, alternative routes of vaccination and boosting of BCG primed immunity *via* viral vectors and protein antigens.

Continued passaging of the original strain in various laboratories across the globe has resulted in a multitude of genomic changes such as single nucleotide polymorphisms, insertions and deletions and the emergence of numerous BCG sub-strains with variable protective efficacy (6). Genomic analyses and transcriptional and proteomic profiling have produced a comprehensive map of all such changes in these sub-strains, that have in turn been classified into groups I-IV (7). Zhang et al. (8), performed a head-to-head comparison of the safety and protective efficacy of 13 BCG sub-strains in mice and showed that the most virulent BCG sub-strains were from group IV (BCG-Phipps, BCG-Pasteur, BCG-Frappier and BCG-Tice) and the least virulent sub-strains were from group II (BCG-Sweden and BCG-Birkhaug). In a study on infants from Uganda, BCG-Denmark induced more scarring and a higher anti-mycobacterial immune response (9) and similar results were reported in another clinical trial that compared BCG-Russia and BCG-Denmark (10). But more recently, a large clinical trial in infants from Guinea-Bissau compared BCG-Denmark, BCG-Russia and BCG-Japan and found that both BCG-Denmark and BCG-Japan were more immunogenic than BCG-Russia (11). Variable efficacy is also driven by other factors such as prior or concurrent infections (non-tuberculous mycobacteria, helminths, parasites), route of vaccination, geographic location (warmer climate, latitude), nutritional status and prior infection with Mtb (12). Although, the exact mechanisms underpinning this variability remain to be established, it seems clear that there are many host effector mechanisms that are poorly stimulated by BCG and that can therefore be exploited as avenues for improvement. Recombinant BCG, BCG prime – boost regimens and the use of non-traditional routes of administration are being explored as strategies to augment vaccine-mediated protection in both uninfected subjects and infected subjects who do not show signs of active disease (also known as latent TB infection (LTBI)). In this review, first we briefly explore the different immune components that may contribute to immunity after BCG vaccination. Next, we summarize current efforts to improve the protective efficacy of BCG efficacy against Mtb through the development of recombinant strains, prime-boost regimens and the exploration of non-traditional routes. Finally, we discuss the potential benefits of BCG for broadly stimulating more generalized host immunity in other diseases such as COVID-19.

## 2 BCG induced innate and adaptive immunity

The recent discovery based on an IFN-γ release assay (IGRA) that BCG protects against Mtb infection and not just progression from LTBI to TB disease (13, 14), has significant research implications as it proves that innate, adaptive as well as trained memory responses are all involved in BCG mediated protection. A strength of the BCG vaccine is that it induces immune responses to a broad range of mycobacterial antigens and requires no additional adjuvants for this immunogenicity (5). These immune responses commence at the inoculation site in the skin where neutrophils, macrophages and dendritic cells interact with the bacterium immediately (15). After the internalization of BCG, dendritic cells mature into potent antigen presenting cells with increased expression of co-stimulatory molecules such as CD80 and CD86, present the antigenic peptides on major histocompatibility (MHC) class II molecules and prime T cells located in lymphoid tissues (16, 17). Dendritic cells stimulate a CD4 T cell adaptive immune response and drive T helper type 1 (Th1) differentiation *via* IL-12, but they also drive a CD8 T cell response through cross presentation of antigens by MHC class I (18). BCG induced macrophage activation also has significant impact on the anti-mycobacterial immune response and studies in mice have shown that mycobacterial killing by macrophages can be observed as early as 7 days post-BCG vaccination, suggesting that a portion of macrophage effector functions may be independent of adaptive immunity (19). Vaccinated mice had a higher percentage of CD11b+ F4/80+ monocyte subset recruitment into the lungs and both macrophages and neutrophils played an important role during the early inflammatory response to reduce mycobacterial burden. A murine intravenous BCG vaccination study showed that macrophages from BCG-vaccinated mice had stronger *ex vivo* control of Mtb growth compared to naïve macrophages, in the absence of B and T cells (20). But neutrophils form the first line of defense and although studies using whole blood have shown that the BCG-induced innate response is driven by monocytes and natural killer (NK) cells (21), neutrophils comprise the majority of cells at the site of BCG immunization (22).

Studies on BCG vaccine immunology have thus far relied heavily upon the hypothesis that polyfunctional CD4 T cells and IFN-γ are the major determinants of its protective efficacy against TB. Earlier studies showing extreme susceptibility of mice with targeted deletion of the IFN-γ gene triggered the evaluation of this cytokine as a possible correlate of protection against TB (23). More recently, Derrick et al. (24), detected polyfunctional CD4 T cells as the predominant T cell population 2 and 8 months after BCG vaccination, but were unable to detect these cells at 14 months, suggesting that CD4 T cells may not persist long-term and therefore likely do not correlate with protection. BCG vaccinated IFN-γ-deficient mice exhibited significant protection against Mtb, suggesting further that CD4 T cells possess IFN-γ-independent mechanisms to limit Mtb (25). Finally, Barber and colleagues showed that IFN-γ accounts for only ~30% of CD4 T cell-dependent bacterial control in the lungs and increasing the IFN-γ-producing capacity of CD4 T cells exacerbated lung pathology with decreased survival (26). In BCG-vaccinated infants in South Africa, the frequency and cytokine profile of mycobacteria specific T cells did not correlate with protection (27) and a systematic review confirmed that despite inducing significantly varied magnitudes of T cell responses in neonates, different BCG strains did not confer different levels of protection (12). These studies and a recent review (28) suggest that, despite their highly relevant role in contributing to immune control of Mtb infection, polyfunctional T cells and IFN-γ could be a measure of the inflammatory response but do not necessarily represent a reliable correlate of protection for vaccines against TB.

### 2.1 Alternate functional subsets of CD4 T cells

While the importance of CD4 T cells with Th1 or polyfunctional properties is well established, other properties or differentiated functions of CD4 T cells have also been implicated in the immune response to BCG vaccination. Vaccines in general rely on the generation of memory T cells to be protective, which results from the clonal expansion and differentiation of antigen specific lymphocytes (29). Central memory T cells (T_CM_) in lymphoid tissues constitute CD4 T cells that maintain a high proliferative capacity, while effector memory T cells (T_EM_) present in the lungs or other nonlymphoid tissues have a high cytokine secretion capacity (30). This suggested that the failure of BCG vaccination to protect adults from pulmonary TB could reflect its failure to induce significant central memory T cell responses (31). Recent interest in mucosal vaccination is in part driven by the discovery of tissue resident memory CD4 T cells (T_RM_) contributing to early clearance of Mtb (32). BCG vaccinated mice were protected against Mtb infection even when egress of cells from the secondary lymphoid tissues was blocked, suggesting that memory T cells generated in the lungs following vaccination were sufficient for protection (33). Mucosal BCG vaccination of mice generated high levels of T_RM_ cells after aerosol Mtb infection and it was confirmed that BCG induced these cells by blocking egress of T cells from the lungs or lymph nodes (34). Intratracheal and intranasal BCG vaccination generated T effector memory and T_RM_ cells in the lung, and adoptive mucosal transfer of these airway-resident memory T cells into naive mice mediated protection against Mtb (35). A recombinant strain of BCG that improved the induction of antigen specific memory T cells provided superior protection compared to standard BCG, and adoptive transfer of the T_CM_ cells in this model further validated their critical role in protection (36). These studies suggest a key role for mucosal vaccination-induced airway-resident T cells in the defense against Mtb and have important implications for the design of more efficacious vaccines.

A suboptimal Th17 response to BCG has been attributed to its lack of the RD1 region, as the RD1-encoded ESAT-6 is a potent inducer of Th17 cells. Adoptive transfer of Th17 cells specific for ESAT-6 partially inhibited Mtb growth, thereby uncovering a previously unrecognized IFN-γ/TNF-α independent pathway. Improved Th17 mediated protection is seen when the RD1 region is restored to BCG by genetic complementation (37). However despite its lack of ESAT-6 secretion, BCG appears to retain some ability to generate Th17 responses that may be influenced by the route and level of vaccine exposure (38). For example, murine studies have shown that IL-17 is produced immediately after pulmonary BCG infection, and impaired granuloma formation has been observed in the lungs of IL-17-deficient mice after aerosol BCG challenge (39). BCG-specific Th17 cells in the lungs are important for optimal Th1 cell recruitment after Mtb challenge (40). A transcriptomic analysis in mice comparing BCG vaccinated and naive mice before and after *M. bovis* challenge found a Th17-related gene expression profile that was predictive of vaccine success (41). RAG-deficient mice (lacking both B cells and T cells) reconstituted with BCG-specific Th17 cells from immunized IFN-γ-deficient mice had better survival and reduced bacterial burdens as compared to RAG-deficient mice that received naïve T cells (38). Furthermore, mucosal BCG vaccination of macaques conferred sterilizing immunity upon Mtb challenge in some animals, and this was significantly correlated with the presence of polyfunctional Th17 cells (42). All of these findings together strongly point to a significant role for Th17 cells in contributing to protective immunity following BCG vaccination, although the induction of Th17 cells may be suboptimal with standard BCG vaccination regimens.

### 2.2 Beyond CD4 T cells: Other T cell effectors of BCG induced immune responses

It is now generally accepted that CD8 T cells have a significant role in protective immunity against Mtb, and the failure of BCG to provide adequate and lifelong protection against TB may be related in part to the insufficient generation of a CD8 T cell response (6). In a murine study that compared the efficacy of oral and systemic routes of vaccination, protection correlated best with the rapid accumulation of CD8 T cells in the infected tissues, whereas the accumulation of CD4 T cells reflected the bacillary load rather than protective efficacy (43). Other animal studies have also suggested that CD8 T cells are important for the control of Mtb infection (44) and could be of particular importance at later stages of infection (45). A recent study showed that both mycobacteria-specific CD4 and CD8 T cells accumulated in the lung after Mtb infection or BCG immunization (34). But other studies have cast doubt on the importance of CD8 T cells during BCG induced immunity against TB. For example, a recent study found that intranasal BCG vaccination conferred superior protection in the lungs with an increased frequency of antigen-specific tissue-resident CD4 T cell response, but no CD8 T cell response was noted in either the spleen, lungs or bronchoalveolar lavage fluid (BALF) (46). Overall, an impression remains that BCG vaccination is relatively poor at inducing CD8 T cell responses, and this is viewed as an area for improvement of modified forms of BCG.

Several other types of unconventional T cells, including but not limited to γδ T cells, Mucosal associated invariant T (MAIT) cells and CD1-restricted T cells, have been implicated in BCG induced immunity (47). For example, increased production of IFN-γ, TNF-α and granulysin was observed from MAIT cells activated by BCG (48). These unconventional T cells are enriched in the respiratory tract and demonstrate antimycobacterial responses and IFN-γ production upon co-culture with macrophages infected with BCG (49). In addition, in a human cohort, primary BCG vaccination was associated with an increase in the γ8.PNG T cell subsets (50). BCG has also been shown to activate innate lymphoid cells (ILCs), which are not T cells but are enriched in the lungs and lymph nodes and may be a significant source of IFN-γ (51).

### 2.3 B cells and antibody responses to BCG vaccination

**Figure 1:**
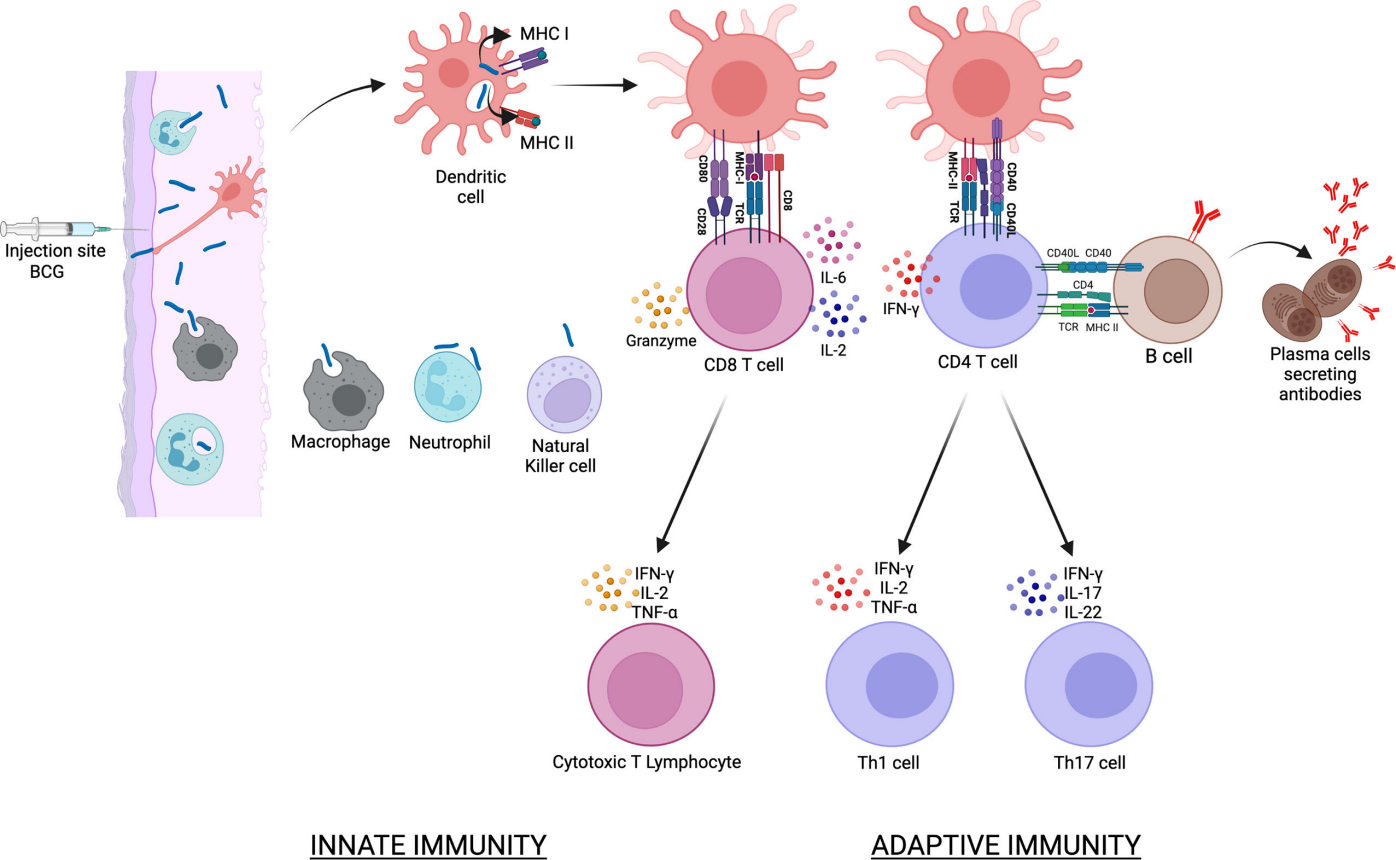
illustrates the events that follow the intradermal injection of BCG. BCG is phagocytosed by various cells of the innate immune response that includes dendritic cells, macrophages and neutrophils. Dendritic cells are the main antigen presenting cells and BCG antigens are presented to CD4 *via* the MHC class II molecules and to CD8 T cells *via* the MHC class I molecules. This is followed by the activation of B cells, cytotoxic T lymphocytes and Th17 cells that collectively constitute the adaptive immune response to BCG vaccination.

Since Mtb is an intracellular organism, the focus on the humoral responses against it has been limited and systematic investigations into B cell and antibody contribution to BCG-induced protection have not been undertaken (52, 53). A recent review by Tanner et al. (53), concluded that the current evidence for BCG driven B cell and antibody responses playing a significant role in protective immunity is inconsistent. *In vitro* studies suggest that antibodies can directly protect through increasing phagocytosis and phagolysosome formation or bacterial neutralization, and indirectly through enhancing T cell-mediated and macrophage mediated killing of Mtb (54, 55). Antibodies from individuals with LTBI have distinctive Fc-mediated functional profiles, potentially mediated by selective binding to different Fcγ receptors and distinct glycosylation patterns (55). Studies in mice have found that intranasal BCG immunization induces secretion of Mtb-specific IgA in the lungs in an IL-17A-dependent manner, which may be associated with reduced bacterial loads in the lungs following Mtb infection (56). In non-human primates (NHPs), intravenous BCG vaccination before Mtb challenge has been found to result in superior protection and higher levels of plasma and BALF IgG, IgM and IgA antibodies specific for the Mtb whole cell lysate (57). Also, in an NHP model, Dijkman et al. (42), compared the standard intradermal and endobronchial route of BCG vaccination and demonstrated a significant increase in PPD-specific serum immunoglobulins following BCG vaccination by both routes. However, IgA was increased by more than 1 log in the BALF following mucosal compared to intradermal administration, and the mucosal route was also able to prevent infection following repeated low dose Mtb challenge. In another NHP study, intravenous BCG drove superior antibody responses in the plasma and lungs of rhesus macaques compared to the traditional intradermal route and the IgM titers negatively correlated with Mtb burden (58). In a combined human and NHP study, BCG-driven induction of specific antibodies was shown to be comparable across humans and macaques, both in magnitude and the range of mycobacterial fractions (59). The study also showed the rapid and transient induction of antibody-secreting plasmablasts following BCG vaccination and *via* the use of *in vitro* functional assays identified a potential FcγR-mediated contribution of antibodies to the control of mycobacterial growth. Collectively, these data suggest the potential importance of antibody responses as a marker and mediator of BCG driven protection against TB. **Figure 1** illustrates the events that follow the intradermal injection of BCG. The presentation of BCG antigens to CD4 and CD8 T cells by dendritic cells is followed by the activation of B cells, cytotoxic T lymphocytes and Th17 cells.

## 3 Evaluation of new modified BCG vaccines and regimens

A significant hurdle in the development of a safe and efficacious TB vaccine has been the lack of an animal model that could accurately reflect the heterogenous nature of the human disease. Generally speaking, the mouse is used for screening TB vaccines as it has a low maintenance cost, a fully defined genetic background and readily available reagents that can delineate complex immunological processes (60). BCG is the gold standard control vaccine and control of infection is said to be achieved with reductions in tissue counts of viable bacilli by at least one log. The general expectation therefore is that any candidate vaccine that aims to replace or boost BCG would provide an improvement on BCG. To progress from the preclinical phase into a clinical trial, vaccine candidates need to be efficacious in at least two animal models, the assumption being that cross-species protective efficacy maybe be reproducible in humans (61). NHPs are phylogenetically closer to humans and are providing critical insights for TB vaccine development (42, 57, 58). Generally speaking, animal studies have not generated a clear consensus on important aspects of a vaccination strategy such as route, regimen and the BCG strain to be used; mainly due to a lack of standardization in the study protocols such as vaccination-to-challenge schedule, route and dose of Mtb challenge and the immune compartments to be studied. This has confounded the interpretation of studies and prevented a direct comparison of vaccines (62). It is therefore important to agree on a standard set of parameters that could allow a meaningful comparison and a reproducible result.

### 3.1 Recombinant BCG (rBCG)

**Figure 2:**
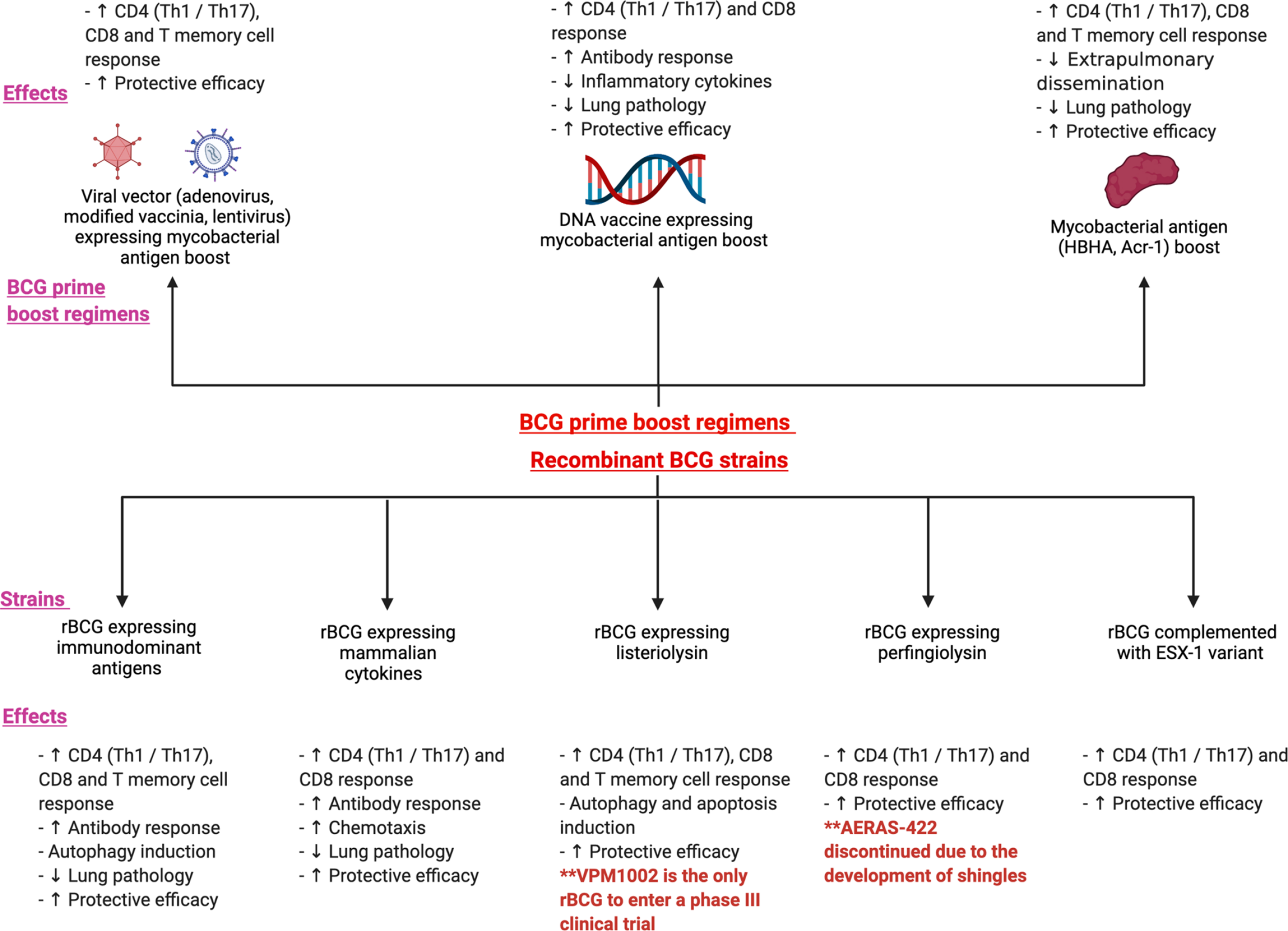
illustrates the various prime boost regimens and the recombinant BCG strains that have been investigated in both animal models and human studies. The figure shows the immunological and protective effects following 3 main BCG prime -boost regimens: viral vector expressing mycobacterial antigen boost, DNA vaccine expressing mycobacterial antigen boost and mycobacterial antigen boost. The bottom panel of the figure depicts the various recombinant strains such as recombinant BCG expressing immunodominant antigens, mammalian cytokines, listeriolysin, perfingiolysin and the ESX-1 variant. Their immunological and protective effects have also been shown.

It has now been three decades since the first studies on the recombinant BCG platform. In the development of rBCG’s, strategies deployed have been the expression or over-expression of antigens critical for mycobacterial physiology (such as IFN-γ and TNF-α), the restoration of antigens that were deleted during the *M. bovis* attenuation process or the inclusion of antigens present only in Mtb. The latter includes immunodominant antigens such as those from the Ag85 family, antigens from the regions of difference RD1 to RD16 and latent phase antigens associated with dormancy and stress resistance (62). rBCG has several advantages over other novel vaccines in terms of lower cost of production, easy storage and an established safety profile. It retains several advantages of the parental BCG such as the ability to protect from central nervous system and childhood TB. In this section, we review the protective correlates of recombinant BCG vaccines expressing immuno-dominant antigens, mammalian cytokines, perfingiolysin, listeriolysin and the ESX-1 variants. **Figure 2** illustrates the various recombinant BCG strains that have been investigated in both animal models and human studies. The various strains and their immunological effects have been shown as well.

#### 3.1.1 rBCG expressing immunodominant antigens

Ag85A, Ag85B and Ag85C comprise major fractions of the secreted proteins in Mtb and are a family of immunodominant antigens with mycolyltransferase activity (63, 64). The rBCG-30 vaccine over-expressing the Ag85B antigen induced protection from Mtb in mice and guinea pigs but clinical trials did not progress beyond phase 1 due to the presence of an antibiotic resistance marker (65). The overexpression of PhoP-PhoR, which regulates the expression and secretion of several genes in the Ag85 family also improved immunogenicity and had a protective efficacy in the mouse and guinea pig models (66). Pym et al. (67), complemented BCG with a construct containing the *esxA* and *esxB* genes, which encode ESAT-6 and CFP-10 respectively. This led to restoration of the RD1 locus and improved protective efficacy after Mtb challenge. Splenocytes from mice inoculated with this vaccine had a high proliferation rate and produced IFN-γ in response to both ESAT-6 and CFP-10. Another recombinant vaccine expressing a fusion protein comprising of Ag85A and ESAT-6 was able to induce higher titers of antibodies and elicit a longer-lasting and stronger Th1 cellular immune response than the parental strain (68). BCG expresses only a small amount of the heat shock protein HspX (latency gene) and induces poor cell-mediated immunity against latency antigens (69). Balb/c mice vaccinated s/c with a recombinant BCG strain overexpressing HspX and Ag85B had a significant increase in antigen-specific IFN-γ to both proteins and showed lower bacterial load and lung pathology than the control mice (70). In another study, a cocktail of recombinant BCG’s named ABX was produced by combining rBCGs that expressed antigens from different stages of the immune response: Ag85A, Ag85B and HspX (71). S/C vaccination with ABX was more protective than parental BCG or the individual antigen rBCGs and this was attributed to a stronger antigen-specific (Ag85A, Ag85B or HspX) CD4 Th1 response and higher numbers of both IFN-γ^+^ CD4 T_EM_ (effector memory) cells and IL-2^+^ CD8 T_CM_ (central memory) cells. The cytotoxic T lymphocyte (CTL) activity was also higher in the ABX group. A recombinant BCG expressing Rv2645 (from the region of difference RD13) improved the antigen presentation capacity of dendritic cells and elicited stronger Th1 and Th17 responses, with fewer Treg cells in mice (72). BCG::Rv2645 exhibited enhanced protective efficacy against H37Rv challenge in both mice and NHPs and transcriptomic analysis revealed an increase in the expression of Th1- and Th17-related genes. A recombinant BCG over-expressing an autophagy-inducing and TLR-2 activating C5 peptide from the CFP-10 protein in combination with Ag85B induced robust antigen presentation to CD4 T cells *in vitro* and elicited strong Th1 cytokines (73). BCG^85C5^ also induced LC3-dependent autophagy in macrophages and expanded both T_EM_ and T_CM_ cells in mice, with protective efficacy against both primary Mtb infection as well as reinfection.

#### 3.1.2 rBCG expressing mammalian cytokines

Another strategy involves expression of cytokines and immunomodulatory molecules that augment the immune response to BCG. The *ifn-γ* and *ag85b* genes were inserted into a Mycobacterial-*E. coli* shuttle vector pMV361 and a novel vaccine expressing both Ag85B and IFN-γ was generated (74). When tested in C57BL/6 mice, it enhanced IFN-γ levels, nitric oxide levels, antigen-specific splenocyte proliferation and the antibody response was skewed towards a Th1 response. In a study that used an rBCG expressing Ag85B, CFP-10 and Interleukin-12 in C57BL/6 and C3H/HeJ mice (75), rBCG vaccinated mice had more IFN-γ-releasing cells in the spleen and lung homogenates as well as a higher Th1 antibody response. Another study that investigated the same vaccine found a higher proportion of polyfunctional CD8 T cells (76) and more pronounced T cell mediated killing of infected macrophages. In a study on BALB/c mice vaccinated subcutaneously with a rBCG expressing a fusion protein of the human IL-12p70 and ESAT-6, the rBCG drove a higher Th1 antibody response, increased CD4 and CD8 T cells in the spleen and was associated with a reduced bacterial burden and reduced pathology in the lungs and spleen (77). C57BL/6 mice vaccinated intraperitoneally with a recombinant BCG secreting a fusion protein of Ag85B and IL-15 demonstrated a robust protection in the lungs after Mtb challenge (78). IL-15 has an important function in the maintenance of memory CD8 T cells and the levels of MHC class Ia and class Ib- binding and IFN-γ producing CD8 T cells were higher after immunization with rBCG-Ag85B-IL15 than after immunization with rBCG secreting Ag85B only. BCG expressing the dominant negative mutant of SOCS1 (suppressor of cytokine signaling molecule 1 molecule) enhanced dendritic cell activation and T cell responses, increased the secretion of IFN-γ/TNF-α/IL-6 and increased protection against Mtb challenge (79). Finally, mice vaccinated with a strain of BCG that secreted high levels of murine monocyte chemotactic protein 3 (MCP-3) displayed increased lymphocyte migration *in vivo* and augmented antigen-specific T-cell responses. The level of protection afforded by BCG-MCP-3 was equivalent to that with control BCG but immunodeficient mice vaccinated with this BCG strain survived significantly longer, thus highlighting it as a safer alternative in immunocompromised hosts (80). Finally, in a guinea pig model, an rBCG overexpressing the endogenous mycobacterial diadenylate cyclase gene and releasing high levels of the STING (stimulator of interferon genes) agonist bis-(3’-5’)-cyclic dimeric adenosine monophosphate (c-di-AMP) reduced lung pathology and CFU scores (81). BCG-*disA*-OE also elicited significantly stronger TNF-α, IL-6, IL-1β, IFN regulatory factor 3 and IFN-β levels than did wild type BCG in murine macrophages.

#### 3.1.3 rBCG expressing listeriolysin and perfringolysin

VPM1002 or BCG *ΔureC::hly* is an rBCG that expresses the *Listeria monocytogenes (L. monocytogenes)* protein listeriolysin O (LLO) instead of urease C. LLO is a cholesterol-binding, pore-forming protein that allows the escape of *L. monocytogenes* from the phagosome and requires a stringent acidic pH of 5.5 for optimal activity. Hence LLO is only active in an acidified phagosome and once in the cytosol, it is recognized by the ubiquitination system and rapidly degraded because of its PEST (proline, glutamate, serine and threonine) sequences (82). The deletion of urease C allows for a partial reversal of the BCG neutralizing capacity and thus for acidification and phagolysomal fusion (83). Expression of LLO in the rBCG leads to perturbation of the phagosome and leakage of bacterial DNA into the cytosol, triggering increased autophagy and apoptosis (84). All these events collectively enhance availability of the bacterial antigens to the MHC class I pathway, ultimately leading to an increase in CD8 T cells (85). BCG *ΔureC::hly* induced more antigen-specific CD4 memory T cells than BCG, with a CXCR5+CCR7+ phenotype (36). A superior protective efficacy of BCG *ΔureC::hly* in mice compared with BCG correlated with the higher proportion of T_CM_ cells and T follicular helper cells. Because it is attenuated, BCG *ΔureC::hly* has been found to be safer in both immunocompetent and immunocompromised mice (83, 86). Phase I/II clinical trials have demonstrated its safety and immunogenicity in humans, including neonates, and a Phase II/III clinical efficacy trial against recurrent TB is ongoing (87). Other clinical trials have shown that it increases IL-17 producing CD8 T cells (88, 89) and in animal studies, a higher proportion of multifunctional T cells positive for IL-2, IFN-γ, IL-17 and TNF-α were also noted. There are great expectations from VPM1002, the only rBCG-based vaccine that has progressed to phase III clinical trials (87). In **Table 1**, we summarize all the clinical trials that are either recruiting or have been recently completed using VPM1002.

**Table 1:** VPM1002 clinical trials in the fight against Tuberculosis.

Study/NCT number	Phase	Sponsor	Participants	Status
Dose-escalation study on the safety and immunogenicity of VPM1002 in comparison with BCG in healthy male volunteers. NCT00749034.	Phase I	Vakzine Project Management GmbH	80. Adult males (18-55 years)	Completed (88, 90)
Dose-escalation study on the safety and immunogenicity of VPM1002 in comparison to BCG in healthy volunteers in South Africa. NCT01113281.	Phase I	Vakzine Project Management GmbH	24. Adults (18-45 years)	Completed
Study to evaluate the safety and immunogenicity of VPM1002 in comparison with BCG in HIV-exposed/HIV-unexposed newborn infants in South Africa. NCT02391415.	Phase II	Serum Institute of India	416. Newborn infants up to 12 days	Completed (91)
Study to evaluate the safety and immunogenicity of VPM1002 in comparison with BCG in newborn infants in South Africa. NCT01479972.	Phase II	Vakzine Project Management GmbH	48. Newborn infants up to 8 days	Completed (89)
Study to evaluate the efficacy and safety of recombinant BCG vaccine VPM1002 in the prevention of recurrent TB. NCT03152903.	Phase II and Phase III	Serum Institute of India	2000. Adults (18-65 years)	Recruiting
Evaluation of the efficacy and safety of VPM1002 in comparison with BCG in the prevention of TB infection in infants. NCT04351685.	Phase III	Serum Institute of India	6940. Newborn infants up to 14 days	Recruiting

Illustrates the various clinical trials with VPM1002 against TB. The table summarizes the study details, NCT number, the phase of the study, sponsor, number of participants, the demographic details of participants and finally the status of the trial (recruiting or completed).

A similar approach was used to generate an rBCG expressing the cholesterol-binding cytolysin perfringolysin O (Pfo), AERAS-401 (92). Generation of this BCG strain (BCG1331 *ΔureC:ΩpfoAG137Q)* was achieved by replacing the *ureC* gene with the *pfoAG137Q* gene under the control of the Ag85B promoter (92). AERAS-401 secretes biologically active Pfo and has a good safety profile in immunocompromised SCID mice. A second-generation derivative AERAS-422 has genes encoding for Ag85A, Ag85B and Rv3407 and expresses the mutant perfringolysin (92). AERAS-422 enhanced immune responses in both mice and guinea pigs compared to BCG and mice immunized with AERAS-422 had better survival after Mtb HN878 challenge (92). In a phase I randomized controlled, first-in-human clinical trial, AERAS-422 induced Ag85A and Ag85B specific lymphoproliferative responses and anti-mycobacterial activity in whole blood bactericidal activity culture assays but the study had to be discontinued as it was associated with the development of shingles (93).

#### 3.1.4 rBCG complemented with ESX-1 variants

BCG lacks the *esx-1* locus which results in sufficient attenuation to allow its use as a vaccine (94). As such there are several immune detection systems that are underused by the vaccine and these offer avenues for the induction of a more potent protective response. ESX-1 is involved in host-pathogen interactions and facilitates the detection of bacterial antigens by cytosolic sensors, thereafter triggering phagosomal rupture and host cell death. But insertion of the Mtb *esx-1* locus into BCG led to increased virulence in immunodeficient mice and prolonged persistence in immunocompetent mice (67). This issue was addressed by inserting the *esx-1* locus of *Mycobacterium marinum*, which has reduced virulence, into BCG (95). Mice vaccinated with BCG::ESX-1^Mmar^ had a higher proportion of CD8 T cell effectors against mycobacterial antigens and ESX-1-specific polyfunctional CD4 Th1 cells. S/C vaccination with BCG::ESX-1^Mmar^ conferred superior protection relative to parental BCG against highly virulent Mtb strains.

### 3.2 Heterologous BCG prime-boost vaccination models

Heterologous prime-boost immunization is a highly effective method for enhancing humoral and cellular immunity. It involves priming the immune system against a target antigen and subsequently boosting these immune responses with an immunogen expressing the original antigen, resulting in synergistic augmentation of immunity (96, 97). This could be characterized by an increased number of antigen-specific T cells, selective enrichment of T cell avidity and increased protective efficacy against the pathogen (98, 99). Here we have summarized key findings and study designs in both animal models and human studies. **Figure 2** illustrates the various prime boost regimens that have been investigated in both animal models and human studies. The figure shows the immunological and protective effects following 3 main BCG prime -boost regimens: viral vector expressing mycobacterial antigen boost, DNA vaccine expressing mycobacterial antigen boost and mycobacterial antigen boost.

#### 3.2.1 BCG prime and viral vector boost regimens

Recombinant adenoviruses (Ad) are considered the most potent T cell immune boosters whereas replication defective vectors such as the modified vaccinia virus, Ankara strain (MVA) are effective in minimizing anti-vector immunity (100). Ad and MVA are both double stranded DNA viruses and have been extensively studied as viral vectors for TB. They are relatively easy to deliver *via* the parenteral or mucosal routes and have inherent adjuvant effects, leading to the induction of immune responses that drive anti-mycobacterial activity (101). MVA vectors are replication-deficient, non-integrating and stably express encoded vaccine antigens (102) whereas Ad are natural mucosal immunogens with an excellent safety record (103). In a seminal study reported in 2003 (104), recombinant MVA expressing Ag85A (M.85A) strongly boosted BCG-induced, Ag85A-specific CD4 and CD8 T cell responses in mice. Intranasal boosting of BCG afforded increased levels of protection in both lungs and spleen and this correlated with the induction of Ag85A-specific, IFN-γ-secreting T cells. In human studies, volunteers primed with BCG prior to AERAS-402 (a recombinant, replication-deficient adenovirus expressing Ag85A, Ag85B and TB10.4) boosting had a significant polyfunctional CD4 and CD8 response with increased lymphoproliferative capacity (105, 106). Kaveh et al. (107), reported a BCG prime boost study using an Ad vaccine expressing the mycobacterial antigen TB10.4 and demonstrated that parenteral boost of BCG immunized mice induced IFN-γ+ CD8 T cells *via* the synergistic priming of new epitopes and an increased breadth of epitope recognition. This induced significant improvement in pulmonary protection against *Mycobacterium bovis*. In a mouse model comparing the protection offered by parenteral and mucosal booster immunizations following subcutaneous BCG priming, protection conferred by BCG was significantly boosted when AdAg85A was given intranasally and correlated with the number of IFN-γ+ CD4 and CD8 T cells in the airway lumen (108). In a study comparing the ability of DNA-, protein- and lentiviral vector-based vaccines that express the antigens Ag85B and Rv3425 to boost the effects of BCG, it was found that the lentiviral vector significantly enhanced immune responses, including Th1 and CD8 cytotoxic T lymphocyte responses (109). Spleens of lentiviral vector boosted mice exhibited a more robust IFN-γ, TNF-α and IL-17 response and this was associated with an increased frequency of IFN-γ^+^ CD4 T cells (after PPD and Ag85B-Rv3425 stimulation) and perforin^+^ CD8 T cells. This prime boost regimen also promoted improved control of bacterial replication in the lungs and spleens compared to BCG alone. BCG vaccinated mice, when intranasally boosted with a replication deficient bovine adenovirus vector expressing Ag85B, were better protected against Mtb challenge and this was associated with a robust expansion of CD4 and CD8 effector, central memory and resident memory cells (110). Finally, in a phase 1, double-blind trial, BCG-vaccinated healthy adults were randomly allocated to receive aerosol MVA85A and intradermal saline placebo or intradermal MVA85A and aerosol saline placebo (111). Ag85A-specific CD4 T cells were detected in bronchoalveolar lavage fluid (BALF) cells from both groups and responses were higher in the aerosol group.

#### 3.2.2 BCG prime and DNA vaccine boost

DNA vaccines have been traditionally considered as priming vectors in heterologous prime-boost regimens but drawbacks have been poor immunogenicity in larger mammals, mostly related to transduction efficiency (97, 112). Boosting BCG-primed mice with a DNA vaccine expressing ESAT-6 and Ag85A resulted in increased IgG levels and increased IFN-γ and IL-10 mRNA in the lungs. This was associated with a reduced bacterial load and decreased lung pathology (113). BCG-primed Balb/c mice that were boosted with a DNA vaccine expressing Ag85A had a significantly reduced bacillary load and this was associated with decreased pathology and lower levels of inflammatory cytokines in the infected lungs (114). Superior protection was associated with increased frequency of splenic IL-2 producing CD4 T cells and increased IL-2 production. Repeated vaccination with a chimeric DNA vaccine encoding Ag85A and two copies of ESAT-6 (HG856A) provided modest protection against Mtb challenge and significantly boosted the immune protection obtained from BCG priming in Balb/c mice (115). Enhanced protection was associated with an increase in polyfunctional Th1 CD4 T cell responses and a higher frequency of Mtb antigen-specific IL-2+ CD4 T cells. In a prime boost strategy with a DNA vaccine expressing Ag85A and GM-CSF, activity of cytotoxic T lymphocytes, spleen cell proliferative responses to Ag85A and IFN-γ as well as the specific antibody titer against Ag85A; were all significantly increased when compared to mice vaccinated with BCG or the DNA vaccine alone (116). The vaccine generated sufficient protection against Mtb in the lungs, spleen and liver.

#### 3.2.3 BCG prime and mycobacterial antigen boost

Even though single protein antigens are able to promote strong immune responses, they have not been found to reach levels of protection similar to BCG. One exception to this is the mycobacterial antigen heparin-binding hemagglutinin adhesion (HBHA) protein that has been found to display a level of protection similar to that of BCG (117). HBHA is a surface associated protein involved in the adherence to epithelial cells and is important for extrapulmonary dissemination of Mtb (118, 119). Prime boost immunization with HBHA induced significantly higher levels of protection compared to vaccination with BCG alone (120). Intranasal immunization induced high recall responses and significantly higher levels of IFN-γ was produced from lymphocytes after BCG priming and intranasal HBHA boost. In a similar study, boosting with HBHA produced enhanced protective immunity in the lungs and spleen against Mtb challenge and was associated with HBHA-specific IFN-γ, IL-12 and TGF-β generation (121). Boosting of s/c BCG primed C57BL/6 mice with intranasal HBHA in combination with a cholera toxin (CT) booster enhanced protective immunity against Mtb and led to increased IFN-γ and IL-17 production from HBHA-specific T cells in the lungs (122). Coexistence of Th1 and Th17 cells was noted in the lungs of HBHA plus CT boosted mice after infection with Mtb.

Several proteins of Mtb are expressed during latency, one such antigen is the alpha-crystalline protein 1 (Acr1) or HspX. Acr1 is considered to be a potent vaccine candidate against dormant Mtb (123). Liposomized Acr1 induced enduring protective immunity against Mtb in BCG primed C3H/HeN mice and this was associated with an increase in multi-functional CD4 and CD8 T cells (124). Significant expansion of both central memory and effector memory CD4 and CD8 T cells was also noted. In a guinea pig model, the BCG prime- Acr1 DNA boost regimen conferred robust protection along with a reduced pathology and fewer bacilli in the lungs and spleen and an increased frequency of multi-functional CD4 T cells (125). Finally, in an NHP model, Lin et al. (126), demonstrated that administering a multistage vaccine (H56) with the adjuvant IC31 as a boost to BCG delayed and reduced clinical disease after Mtb challenge, and also prevented the development of LTBI. H56 contains Ag85B, ESAT-6 and the nutrient stress-induced antigen Rv2660c. Boosted animals showed reduced pulmonary pathology and extrapulmonary dissemination, and protection correlated with a strong recall response against ESAT-6 and Rv2660c. Finally, in mice and guinea pigs, boosting BCG primed animals with a complete deletion mutant of the Mtb Esx-5 type VII secretion system (Mtb Δesx-5) improved protection against highly virulent Mtb strains and was associated with increased pulmonary influx of T_CM_ cells, follicular Th cells and lower numbers of T cells expressing exhaustion markers (127).

## 4 Optimal dose of BCG vaccination

Relationship between the dose of BCG administered and the protection achieved has not been well defined. Since the degree of protection from the BCG vaccine has been notoriously unpredictable, the largest available dose of BCG was initially recommended by the WHO. Bretscher et al. (128), proposed that this dose of BCG being given to children was excessively high and was leading to a mixed Th1/Th2 response and that a lower dose could skew this towards a predominantly Th1 response (129). In a murine study with a range of doses of BCG, a Th2 response and higher IgG levels were induced by a higher dose of BCG, but this did not correlate with protection (130). Horwitz et al. (131), showed that high and low doses of a recombinant BCG vaccine expressing Ag85B induced comparable lymphocyte proliferative responses and delayed-type hypersensitivity responses and that a very low inoculum of this rBCG strain had the capacity to induce strong protective immunity against Mtb. Power et al. (132), showed that low doses of BCG generated a relatively exclusive Th1 response and this was independent of whether BCG was given intravenously, subcutaneously (s/c) or intradermally. The study also showed that low dose vaccinated BALB/c mice had better resistance to TB and that the dose of antigen determined the type of antigen presenting cells stimulated. Older studies have shown that the strength of T cell receptor (TCR) signaling can control naïve T cell differentiation, with low antigen doses resulting in weak signaling and a Th2 biased effector cell response whereas strong TCR signaling (either high affinity antigen or high dose) skewing towards a Th1 biased effector cell response (133). This mechanism could at least partially account for some of the BCG dose effects but it is not consistent with studies by Bretscher and colleagues (128, 129), who claimed that low doses of BCG imprinted a Th1 response in mice, while high doses gave a mixed Th1/Th2 response. In a recent study using different mouse strains and BCG at a lower dose of 3000 CFUs per mouse (a human equivalent dose), protective efficacy in BALB/c and CB6F1 mice was demonstrated and such an approach of using human equivalent doses is a realistic and practical for preclinical studies (134).

## 5 Routes of BCG vaccination

BCG is typically administered *via* the intradermal route and this induces a strong systemic response but a weak mucosal response. The resultant failure to generate long-lasting immunological memory in the lungs has been proposed as one of the possible cause for the poor level of protection conferred *via* the intradermal route (135). One explanation for this is that the delivery of BCG by the intradermal or s/c route results in sub-optimal migration of bacilli to the draining lymph nodes and a delayed encounter with primed antigen presenting cells in the lungs. Recent years have seen a resurgence of interest in the mucosal delivery route as it has been shown to induce a better immune response both in the systemic and mucosal compartments.

### 5.1 Mucosal routes

#### 5.1.1 Intrapulmonary BCG vaccination

Organized lymphoid aggregates are present both in the nasal and bronchial mucosa and have an important role in the initiation of mucosa-associated immunity against infectious agents (136, 137). One of the shortcomings of the s/c route is the overall weak memory lymphocyte generation that lacks the mucosal-homing chemokine receptors required for migration to the lungs. Mucosal routes of vaccination are therefore being investigated as a mimic of natural infection with the aim to improve local immunity (138). Murine studies with adoptive mucosal transfer have shown that lung tissue cells associated with a protective phenotype are induced more intensively by mucosal BCG vaccination than *via* the intradermal route (35). A comparative study of the protective efficacy of intratracheal and s/c routes of vaccination with the BCG Danish strain found significantly reduced bacterial counts in mice after intratracheal vaccination and was associated with a higher proportion of IFN-γ^+^ CD4 T cells after *ex vivo* stimulation of splenocytes with PPD (139). A prominent discovery in recent years has been the role of Th17 cells that can be induced by mucosal BCG. The recombinant BCG vaccine *ΔureC::hly* induces increased numbers of Th17 cells and renders better protection than the parental strain (85). In pre-clinical studies of this vaccine, there was an association between vaccine associated protection and CXCR5^+^ CCR7^+^ central memory CD4 T cells (36). Improved BCG induced protection was achieved when the vaccine was given endobronchially to rhesus macaques (140) and mucosal, but not s/c BCG vaccination generated lung-resident memory T cell populations that conferred enhanced protection (35). NHP’s vaccinated with BCG *via* the intratracheal route as a boost to previous intradermal BCG had a reduced level of pulmonary disease after Mtb challenge and endobronchial BCG administered protected NHP’s from repeated low-dose Mtb infection, with a higher proportion of polyfunctional CD4 and CD8 T cells noted in the BALF (141). In addition, granzyme B, IL-10, granulocyte-macrophage colony-stimulating factor (GM- CSF) and IgA levels were higher in the mucosal compartment. Th17 cells, IL-10 and IgA were identified as correlates of protection. Mucosal but not intradermal vaccination, either with BCG or the Mtb-derived candidate MTBVAC, enhanced innate cytokine production by blood- and bone marrow-derived monocytes, typical of trained immunity (142). Besides the ease and safety of administration, the mucosal route has the significant advantage to induce a more protective immune response locally. Aerosol BCG can also induce higher protection against Mtb in rhesus macaques (143, 144) but exact dose delivery and the uniformity of distribution is difficult to control. It is being tested in early-phase clinical trials (NCT02709278) but there are concerns regarding safety as the cell wall lipids of BCG are inflammatory. Moliva et al. (145), selectively removed these inflammatory lipids without affecting bacterial viability and the delipidated BCG evoked an attenuated inflammatory response, suggesting that it could be used safely *via* the mucosal route. Delipidated BCG imparted superior protection than non-delipidated BCG comprising of an increase in Th17 cells and CD4 and CD8 central memory and effector memory cells.

#### 5.1.2 Intranasal BCG vaccination

Uranga et al. (146), have shown that the pulmonary immune response in mice infected with Mtb was improved when BCG was administered intranasally. This was associated with an increased presence of IgA and IL17+ CD4 T cells in the BALF. A greater percentage of antigen-specific IFN-γ producing cells was observed after Mtb challenge in the spleens of intranasally vaccinated Balb/c mice as compared to s/c vaccinated mice (147). Larger number of BAL inflammatory cells were also found and led to better protection from Mtb. In a similar study, intranasal BCG vaccination led to a reduced bacterial burden in the lungs in comparison to s/c BCG vaccination, with a concurrent greater proliferative response and higher levels of IFN-γ and IL-12p40 in the lungs (148). In C57BL/6 mice, intranasal BCG vaccination induced a higher splenic response compared to s/c vaccination with a higher frequency of splenic polyfunctional CD4 and CD8 T cells (149). There was an associated increase in the mRNA expression of IFN-γ, IL-9, IL-11 and IL-21 in the spleen but despite improved protection in the lung at early time points, no difference was noted at 8-10 months post vaccination. In the tuberculosis-susceptible DBA/2 mice, intranasal but not s/c BCG conferred a robust protection against a pulmonary TB challenge and was associated with an Mtb-specific mucosal immune response that was orchestrated by IL-17 (56). IL-17 neutralization *in vivo* reduced protection and abrogated Mtb-specific IgA and BCG-induced expression of polymeric Ig receptors in the lungs. In BALB/c mice, intranasal BCG vaccination conferred superior protection in the lungs compared to the intradermal route, with an increased frequency of antigen-specific tissue-resident CD4+ T cells with distinct phenotypes and an enhanced proliferative capacity (46).

#### 5.1.3 Oral BCG vaccination

The orally delivered BCG substrain Moreau Rio de Janeiro has a long established safety profile through usage up and until the 1970’s in Brazil and through clinical trials in the UK (150). Oral vaccines are easy to administer and bypass the use of needles but have to be robust enough to withstand the low pH in the stomach. Lipid microencapsulation of BCG can extend the *in vivo* survival of BCG when fed to mice and result in long-lasting systemic cell-mediated immune reactivity and a high level of protection from *M. bovis* challenge (151). Oral BCG induced a long-lived multifunctional CD4 T cell response in the lungs, with an increase in Ag85B tetramer specific CD4 T cells in the spleen for up to 30 weeks post vaccination (152). It has been reported though that the immunogenicity and efficacy of orally administered vaccines is lower in the developing world, where helminthic and *H. pylori* infections have been shown to decrease BCG-induced immune responses (153). Since the vast majority of high burden TB areas are in the developing world, this observation makes the trial of an oral TB vaccine hard to justify. Both intrarectal and intragastric BCG vaccinations have been investigated (154, 155) and it was found that an enormous dose of BCG *via* these routes was required to achieve a level of protection comparable to that of intradermal vaccination, thus precluding them as a realistic choice.

### 5.2 Systemic routes

#### 5.2.1 Intralymphatic BCG vaccination

Intralymphatic BCG vaccination of C57/BL6 mice has been shown to be more effective in stimulating BCG-specific immune responses in comparison to the intradermal or subcutaneous routes (156). The intra-lymphatic route stimulates a higher frequency of mycobacterium-specific CD4 and CD8 T lymphocytes with a strong proliferation index and a higher production of IFN-γ, TNF-α, IL-2 and IL-17. It was also shown that the intralymphatic route induced a sustained protection against Mtb challenge whereas s/c vaccination conferred a transient protection only.

#### 5.2.2 Intravenous BCG vaccination

The first intravenous BCG injections were performed by Calmette and Guerin, and were mentioned in Calmette’s book of 1927 (157). Exploring the intravenous route of vaccination was thereafter suggested in the 1970’s (158) and interest was more definitively revived when Sharpe et al. (141), showed decreased lung pathology and improved survival in the NHP model. In murine studies, it has been shown that intravenous BCG induces trained immunity, and epigenetically modifies monocytes and NK cells kill Mtb more effectively (159, 160). In a seminal study with rhesus macaques, Darrah et al. (57), showed that intravenous BCG elicited a high frequency of antigen responsive systemic and tissue resident T cells, with an up-regulation of BALF CD4 T cell genes that were protective against TB. These findings translated into a protective outcome after Mtb challenge, as determined by imaging, CFU measurements and lung pathology. Mechanistically speaking, the authors surmised that the rapid elimination of Mtb maybe due to the high magnitude of Th1/Th17 responses in the lungs and high levels of IgG and IgA in the BALF, although the latter had waned to pre-vaccination levels at the time of Mtb challenge. The Seshadri group (161) have shown that mycobacterial glycolipid specific T cells expand in the blood of NHP’s 4 weeks after intravenous BCG vaccination with a predominant effector memory phenotype. These cells were also present in the lungs as tissue resident memory cells 4 weeks after vaccination and contributed to protective immunity. In rhesus macaques, intravenous BCG enhanced innate cytokine production associated with changes in histone acetylation typical of trained immunity (142). However, direct administration of live BCG bacteria into the bloodstream of people is bound to raise concern, particularly in immunocompromised individuals. Splenomegaly has been noted in NHPs after intravenous BCG administration (57) and raises serious concerns about its utility in humans. Moreover, the safety and efficacy of intravenous BCG in those with prior immune sensitization to mycobacteria, concurrent infection with Mtb or undiagnosed LTBI is also unknown. Thus, the above studies provide support to strategies for improving TB vaccination *via* the intravenous route but further clarification of risks will be required for the intravenous route to gain acceptability in clinical trials.

## 6 BCG revaccination

Nemes et al. (14), evaluated the prevention of quantiferon (QFT) conversion in healthy South African adolescents by means of BCG revaccination or H4:IC31 (subunit vaccine containing Ag85B and TB10.4) vaccination. The study found that vaccination with either agent reduced the rate of sustained QFT conversion in a high-transmission setting, suggesting that clearance of initial infection was occurring in some vaccinated subjects. BCG revaccination had a 45.4% efficacy rate against sustained QFT conversion but the efficacy rate of H4:IC31 (30%) did not meet statistical significance. Previous large trials had shown no benefit *via* revaccination with BCG, but none of these trials had selected on the basis of the status of LTBI/Mtb infection or measured infection acquisition during follow up (162–164). In a placebo-controlled phase 2b trial, 2 doses of the M72/AS01_E_ vaccine (recombinant fusion protein derived from the Mtb antigens Mtb32A and Mtb39A) in LTBI individuals who had been previously vaccinated with BCG, prevented bacteriologically confirmed pulmonary TB (165). In a follow up study, the same vaccine provided approximately 50% protection against progression to active TB for 3 years (166). M72-specific antibodies and CD4 T cells remained elevated during the study duration. Both these trials involved prospective collection and storage of blood and will allow detailed analyses of the full spectrum of anti-mycobacterial immune responses as correlates of protection. Finally, a more recent study demonstrated that BCG re-vaccination of young adults induced Ag85A and BCG-specific CD4 and CD8 T cell responses (167). Polyfunctional Ag85A-, BCG- and Mtb- latency Ag-specific polyfunctional CD4 T cells were also significantly enhanced and the study was the first to show that BCG revaccination boosts antimycobacterial Th1/Th17 responses. However, two recent trials in Africa have shown less promising outcomes with BCG revaccination. The first was a double-blind, randomized, placebo-controlled trial of repeat BCG vaccination and showed that after a 6-9 year follow up, repeat BCG had a 49% efficacy against leprosy but no protection against TB (168). The second study was a population-based, double-blind, randomized controlled trial that enrolled over 45,000 individuals in Northern and Southern Malawi (169). The study showed no beneficial effect of BCG revaccination on all-cause mortality. These discrepant results will stimulate larger epidemiological trials for a more definitive answer. Clinical trials can help consolidate a biosignature of protective immunity that can help design successful revaccination strategies.

## 7 Survival benefits of BCG

Since its introduction in Europe in the 1920’s, there have been reports that BCG reduces infant mortality rates to an extent that could not be explained by a reduction in TB alone. Initial evidence was described in Sweden in the 1920’s when it was found that BCG-vaccinated infants had a mortality rate that was one-third that of unvaccinated infants in the first year of life (170). Besides protecting against tuberculous meningitis and miliary TB, BCG provided non-specific cross –protection against other infectious diseases, especially neonatal respiratory tract infections, malaria and neonatal sepsis (171, 172). A study in Guinea-Bissau showed that the presence of a BCG vaccination scar was associated with diminished mortality due to malaria and respiratory syncytial virus (173) and in a Spanish cohort, BCG vaccination correlated with reduced overall hospitalizations due to respiratory infections from all causes in children under 14 years of age (174). A cluster sampling study of BCG immunization in infants in more than 30 countries revealed a reduction in the incidence of acute lower respiratory tract infections by 17-37% (175). BCG’s effects on decreasing infant mortality in a non-specific manner give it a huge advantage over other vaccines that confer protection only against specific pathogens, and this effect may be life-saving for children who have not yet developed adult levels of adaptive immunity.

There is also evidence suggesting a similar effect on boosting of general immunity by BCG in adults. The phase III ACTIVATE trial was a double-blind, randomized trial, that recruited elderly patients to receive either the BCG or a placebo vaccine at the time of hospital discharge (176). At interim analysis, BCG vaccination significantly increased the time to first infection and reduced the incidence of new infections, mostly those due to respiratory pathogens. BCG has been tested in three randomized controlled clinical trials, one case-control study and four case series for efficacy against viral infections and every study showed a beneficial effect (reviewed in (177)). These randomized controlled trials strongly suggested causality for BCG protection against viral infections. As an example, the high mutation rate of the influenza virus can impair the efficacy of specific vaccines and therefore the broad range of pathogens that BCG can protect against is a huge advantage in the elderly population who are very susceptible to influenza. BCG driven reduction in morbidity and mortality in these studies could be driven by trained immunity, a phenomenon that we discuss in the next section, and since trained immunity has the potential to decrease co-infections, BCG vaccination in children, the elderly and the immune-compromised could be judiciously used to decrease mortality (178). Thus, improving trained immunity could be an ideal complement for the T cell and B cell responses driven by BCG.

## 8 BCG mediated trained immunity

Netea et al. (179), first reported the concept of “trained immunity”, a biological process through which the immune response of innate immune cells is amplified following a previous exposure to unrelated agents, independent of B and T cells (180, 181). The primary mechanisms that govern trained immunity are epigenetic reprogramming and changes in the immune metabolism. During a primary exposure, there are epigenetic changes leading to active transcription of pro-inflammatory cytokines and upon removal of the primary stimulus, the trained cell goes back to a resting state while retaining these epigenetic signatures. With a future second heterologous challenge, there is a pronounced expression of inflammatory mediators and this results in a more successful immune clearance. BCG mediated trained immunity increases the capacity of monocytes and NK cells to protect against organisms such as *Staphylococcus aureus*, *Candida albicans* and experimental yellow fever (182). A randomized controlled trial found that BCG vaccination accelerated the acquisition of influenza (H1N1) antibodies and thereby enhanced the immunogenicity of subsequent influenza vaccination (183). In another study, BCG vaccination decreased yellow fever virus (YFV) viremia after challenge with an experimental YFV strain and displayed higher levels of IL-1β release, which correlated with epigenetic modifications (182). In an attempt to better understand the mechanics of BCG-induced trained immunity, Kong et al. (184), performed single cell transcriptomic measurements with bacterial lipopolysaccharide (LPS) and found that prior BCG vaccination reduced the systemic inflammation caused by LPS. The study also identified age-dependent gene expression changes in monocytes and BCG-trained adult monocytes demonstrated enhanced TNF-a production whereas BCG-trained newborn monocytes demonstrated tolerization and immunometabolic shifts (185). BCG vaccinated TB case contacts without evidence of Mtb infection had higher levels of pro-inflammatory cytokines in response to heterologous stimuli such as *Escherichia coli* and *Streptococcus pneumoniae*, providing further evidence of BCG-induced trained immunity in these individuals (186).

In addition to the effects of BCG-induced trained immunity in tackling infections, there may also be significant effects on inflammation and autoimmunity. Cheng et al. (187), showed that BCG can influence immune metabolism, shifting the balance in cellular metabolism towards glycolysis as the energy source rather than oxidative phosphorylation. This metabolic shift, known as the Warburg effect, favors the expansion and function of Treg cells, which in turn might be useful in several inflammatory conditions. Patients with long-term type 1 diabetes were found to have near-normal levels of Hemoglobin A1c and elevated expression of regulatory Foxp3 cells after 2 doses of BCG (188). These effects were maintained for 5 years. In non-obese diabetic mice, BCG was shown to reduce insulitis (189). In another study, patients with multiple sclerosis receiving BCG had fewer lesions 6 months postvaccination on a brain MRI (190). BCG reduced the severity of experimental autoimmune encephalitis (EAE) in a murine model *via* a reduction of Th17 cells and up-regulation of Treg cells (191). The activated CD4 T cells undergo apoptosis, with a concurrent diminution of autoreactive T cells. The exact mechanisms of these phenomena are yet to be delineated but it is believed that BCG induces a tolerogenic response *via* the enhancement of glycolysis (192).

Effects of BCG in the treatment of cancer have also been attributed at least in part to mechanisms based on trained innate immunity. In a landmark study, Morales et al. (193), treated patients with recurrent non-muscle invasive bladder cancer (NMIBC) with weekly intravesical and intradermal BCG and observed a 12-fold reduction in bladder cancer recurrence. This finding has been confirmed and extended by many subsequent reports, and intravesical instillation of BCG has become the current standard treatment for NMIBC. Recently, potential improvement to this treatment was suggested by a phase 1/2 single arm trial (NCT02371447) using the recombinant BCG vaccine strain VPM1002, in which almost half of the patients with a recurrence after previous conventional BCG were free from NMIBC and this may be in part due to trained immunity (194). In bladder cancer, BCG induces the infiltration of macrophages, T cells and NK cells, implicating potential roles for both adaptive as well as trained innate immunity (195).

**Figure 3:**
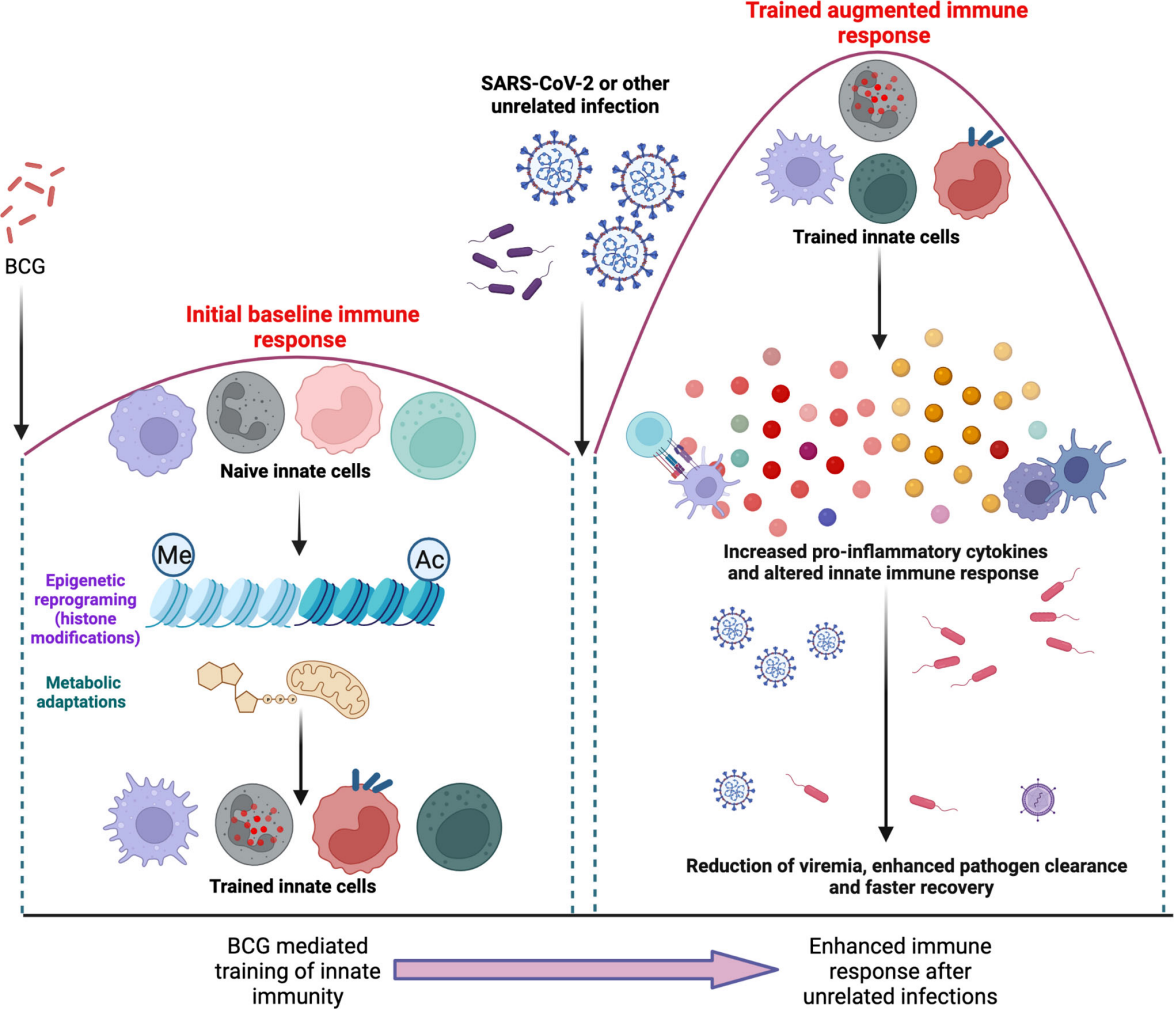
illustrates the “training” of naïve immune cells after BCG vaccination *via* epigenetic reprograming and metabolic adaptations (left panel). Subsequently, upon a future encounter with an unrelated infectious agent, these “trained” cells of the innate immune system are able to mount an altered immune response that is more effective in the reduction of viremia, clearance of pathogens and a faster recovery.

BCG mediated trained immunity has also been implicated in protection against mycobacteria. In some western countries that do not have a BCG vaccination policy, the increase in the rates of non-tuberculous mycobacterial (NTM) infection has renewed the interest in BCG vaccination (196). Murine studies have shown BCG mediated protection against NTM infection (197). Peripheral blood mononuclear cells isolated from BCG vaccinated healthy adults produced increased levels of innate cytokines such as TNF-α in response to Mtb lysate and displayed increased cell surface expression of activation markers (159). Macrophages from BCG vaccinated subjects displayed altered DNA methylation patterns on promoters of immunity-related genes (198) whereas peripheral blood mononuclear cells from BCG vaccinated infants were enriched for expression of various immunity-related genes (199). It has also been shown that human neutrophils undergo long-term immunophenotypic changes after BCG vaccination with enhanced expression of activation markers, increased production of chemokines and enhanced reactive oxygen species production (200). In another study, NK cells from BCG vaccinated healthy adults produced increased levels of pro-inflammatory cytokines upon *ex vivo* stimulation with mycobacteria (180). Finally, the hypothesis that BCG induced trained immunity confers protection from Mtb infection was further supported by the BCG revaccination trials in South Africa that saw a reduction in the risk of sustained blood Quantiferon test conversion by 45% (14). However, the impact of trained immunity in more vulnerable patients such as the elderly or the immunocompromised is not established, and the secondary boosting of innate immune cells with the increased production of IFN-γ and TNF-α could potentially be harmful in these subjects. **Figure 3** illustrates the “training” of naïve immune cells after BCG vaccination *via* epigenetic reprograming and metabolic adaptations. Subsequently, upon a future encounter with an unrelated infectious agent, these “trained” cells of the innate immune system demonstrate a more effective immune response that is helps with enhanced pathogen clearance.

## 9 Implications for the COVID-19 pandemic

Representing another potential benefit of BCG induced trained innate immunity, several ecological and observational studies have proposed a correlation between BCG vaccination policies and COVID-19 related mortality and morbidity (201). Both mortality and crude case fatality rates were lower in countries with a BCG vaccination program; with a significantly lower incidence of COVID-19 cases in countries that had BCG in their national vaccination programs (202). A study using growth curves for confirmed cases of COVID-19 showed a correlation between BCG vaccination and daily rates of COVID-19 cases and deaths in the first 30 day period of a country’s outbreak (203). Subsequently, an assessment of the global linkage between BCG vaccination and COVID-19 mortality showed a strong correlation with the BCG vaccination rate, indicating that for every 10% increase in the country’s BCG vaccination rate (reported as BCG index) there was a 10% reduction in COVID-19 mortality (204). These studies should however be interpreted with caution as they are prone to bias from confounders such as differences in national demographics (health and education), reporting biases, death certification rates, testing rates and the stage of the pandemic (205). Indeed, several subsequent studies have failed to demonstrate a correlation between BCG and SARS- CoV-2 infection. For example, a study that compared COVID-19 mortality by national BCG use pattern did not find any protective influence (206), and a study from Israel using subjects from before and after the discontinuation of universal BCG vaccination failed to identify differences in age-specific COVID-19 protection (207). A 2021 study has attempted to reconcile these conflicting findings by modelling the effect of BCG vaccination across different times during the pandemic, with the conclusion that the protective effect of BCG against COVID-19 was strongest in any particular country during the early stages of the pandemic (208).

Further clinical data to support the hypotheses based on observational and ecological studies is emerging. In a retrospective study, volunteers who received the BCG vaccine within the past 5 years had a lower incidence of self- reported sickness and fatigue compared with those who had never received the vaccine (209). A retrospective observational study of a diverse cohort of health care workers in Los Angeles, California, demonstrated that a history of BCG vaccination was associated with reduced COVID-19–related clinical symptoms as well as a decreased prevalence of anti–SARS-CoV-2 IgG (210). Importantly, no such association was found with meningococcal, pneumococcal or influenza vaccination. Ultimately, these findings need confirmation and several phase III–IV trials are currently underway around the world to obtain more definitive answers. In a murine study, intravenous BCG protected human-ACE2 transgenic mice against SARS-CoV-2 and the phenotype was associated with reductions in tissue pathology, inflammatory cell recruitment and cytokine production that multivariate analysis revealed as only partially related to the diminished viral load (211). A number of interesting and possibly relevant findings on immunological cross-reactivities between BCG and SARS-CoV-2 antigens have been reported. It has been shown that an antibody directed against the SARS-CoV-2 envelope, but not the spike or membrane proteins, strongly cross reacts with several mycobacterial species (212). Sequence analysis using BlastP showed high homology of the SARS-CoV-2 envelope proteins with amino acids of the protein LytR C, a consensus protein unique to mycobacteria. In a study that used BCG-derived peptides, it was shown that 8 peptides had significant sequence homology to SARS-CoV-2 peptides and human CD4 and CD8 T cells primed with these BCG-derived peptides developed enhanced reactivity to corresponding homologous SARS-CoV-2 derived peptides (213). Thus, cross reactivity between BCG vaccine antigens and antigens from unrelated pathogens with shared T- and B-cell epitopes can induce heterologous effects in adaptive immunity (214). Another mechanism is bystander activation, where non-relevant and heterologous T cells with a specificity different from cells involved in the classical immune response are activated without strong T cell receptor ligation, *via* cytokines such as IL-2 driven by the activation of cells during the classical response (215). These findings suggest that both trained and adaptive immunity driven by BCG vaccination may contribute to immunity against SARS-CoV-2.

As of December 2020, several SARS-CoV-2 vaccines have been approved for therapeutic use, including two messenger RNA based vaccines and adenovirus vector-based vaccines. Regardless, important arguments have been made in support of continuing BCG trials for COVID-19 (216). First, BCG may increase the efficacy of SARS-CoV-2 specific vaccines *via* the development of memory T cells and IgA in the lungs, thereby helping to mitigate secondary viral and bacterial infections. Trained immunity can play an important role in decreasing viremia, enhanced pathogen clearance and reduced inflammation. Another avenue *via* which BCG can have an immuno-modulatory effect is through the restoration of Treg cells and the induction of tolerogenic pathways that can dampen dysregulated inflammation seen in COVID- related acute respiratory distress syndrome (ARDS). A combination therapy approach using BCG and drugs that block pro-inflammatory cytokines (such as the anti-IL-6 antibody Tociluzimab) may also be helpful in generating a more regulated immune response in COVID-related ARDS.

Although currently there is no direct evidence to support the use of the BCG vaccine for the prevention or mitigation of coronavirus infection, multiple trials are ongoing as summarized in a recent review (217). Among these, the BATTLE trial in Brazil (ClinicalTrials.gov database #NCT04369794) is a prospective, randomized, double-blind study that is investigating the potential of BCG to affect the clinical evolution of COVID-19 and also the seroconversion rate *via* trained and adaptive responses. Another potential approach is the use of recombinant BCG strains engineered to enhance both innate and adaptive immune responses in populations with high exposure to SARS-CoV-2 (NCT04387409 and NCT04439045). It has been speculated that BCG revaccination or the mucosal BCG can engage the trained immunity locally and lead to more effective immune responses in the lungs (218). The duration of protection conferred by COVID-19 vaccines is unknown and one thought is that a combination of BCG with a COVID-19- specific vaccine might induce longer lasting protection. Finally, global demand for COVID-19 vaccines is likely to continue to outstrip availability in many regions. As BCG is widely available, its use can help bridge this gap.

## 10 Conclusions

Due to the important role of BCG vaccination in reducing infant mortality rates and its non-specific yet important effects *via* trained immunity, several current attempts toward improved TB vaccines are being designed as a heterologous boost to a BCG prime. Thus, BCG will likely continue to be widely used well into the future and it is therefore critical to understand the immune response to BCG as this will help in the design of improved BCG strains and novel prime-boost regimens. A major obstacle in the development of an efficacious TB vaccine has been the lack of consensus on immune correlates that could be a surrogate marker of protective efficacy. Studying the immune responses to BCG offers a valuable opportunity to explore the correlates of protection against TB that can be applied to new vaccine development. Effector functions such as the ability to recognize Mtb, T cell phenotypes and T cell differentiation states will likely need to be included to predict the protective potential of a vaccine. In addition, BCG driven trained immunity offers new avenues, especially if studies show that it correlates with early clearance of Mtb. Given that trained immunity is mediated by epigenetic modulation and changes to intracellular metabolism, incorporating metabolic and epigenetic modulators to amplify vaccine-induced responses is another avenue to be considered.

It is unlikely that a single immune marker can predict protection imparted by BCG, instead it is likely that a combination of host factors assessed by a systems biology multi-omics approach will provide an immune biosignature that would be predictive of protection. Recent progress in high-throughput technology such as genomics, proteomics and metabolomics enable such a big picture view of the immune status as they can study immunological differences between unvaccinated control groups and vaccinated groups. The “omics” technology can overcome the lack of a good human challenge model as it can be used in large heterogeneous cohorts of household contacts to study their protective immunity as a natural control group (219). Key areas to improve on BCG protection against Mtb infection include the duration of protection and protection across different Mtb strains. Novel technologies such as CRISPR can facilitate mycobacterial genome editing to further improve the engineering of recombinant BCG for greater immunogenicity. These technologies allow for in-depth and unbiased profiling of cell populations in animal models and human studies and will be the next chapter in the development of a TB vaccine. Study of the complex and heterogeneous immunological effects of the BCG vaccine offers an unparalleled opportunity to gain insights into the development of a more successful TB vaccine. The COVID-19 pandemic has brought new technologies such as the messenger RNA vaccine into the vaccine arena, and the next decade will likely see increasing efforts to apply these new technologies along with conventional strategies to build better vaccines in the battle against TB.

## Author contributions

SS wrote the 1^st^ draft and all sections of the manuscript. SS made all the figures. NASA and ST helped with all sections of the manuscript and provided valuable insight with the questions we discuss in this review. SP gave overall direction and vision to the manuscript, helped with all subsequent drafts and edited all figures. All authors contributed to the article and approved the submitted version.

## Funding

Stony Wold Herbert Inc. Foundation and ATS COPD Foundation Bronchiectasis Initiative Grant (SS), NIH SC1GM140968 and BBRC (ST), R01 AI137344, AI127711 and AI045889 (SP).

## Acknowledgments

Figures were created with BioRender.com.

## Conflict of interest

The authors declare that the research was conducted in the absence of any commercial or financial relationships that could be construed as a potential conflict of interest.

## Publisher’s note

All claims expressed in this article are solely those of the authors and do not necessarily represent those of their affiliated organizations, or those of the publisher, the editors and the reviewers. Any product that may be evaluated in this article, or claim that may be made by its manufacturer, is not guaranteed or endorsed by the publisher.
